# Aqueous Extracts of* Cordyceps kyushuensis* Kob Induce Apoptosis to Exert Anticancer Activity

**DOI:** 10.1155/2018/8412098

**Published:** 2018-08-09

**Authors:** Xuan Zhao, Xing-hui Yu, Guo-ying Zhang, Hai-ying Zhang, Wei-wei Liu, Chang-kai Zhang, Ying-jie Sun, Jian-ya Ling

**Affiliations:** ^1^State Key Laboratory of Microbial Technology, Shandong University, Jinan 250100, China; ^2^Shandong University of Traditional Chinese Medicine, Jinan 250014, China; ^3^Dezhou University, Dezhou 253023, China

## Abstract

Cancer has become the leading cause of mortality since 2010 in China. Despite the remarkable advances in cancer therapy, a low survival rate is still a burden to the society. The antineoplastic activity of aqueous extracts of* Cordyceps kyushuensis* Kob (AECK) was measured in this study. Results showed that AECK can significantly inhibit the proliferation and viability of U937 and K562 when treated with different concentrations of AECK, and the IC_50_ values of U937 and K562 were 31.23 *μ*g/ml and 62.5 *μ*g/ml, respectively. Hoechst 33258 staining showed that AECK could cause cell shrinkage, chromatin, condensation, and cytoplasmic blebbing, and DNA ladder experiment revealed the evident feature of DNA fragmentation which showed that AECK could induce cell apoptosis. Moreover, AECK gave rise to intrinsic apoptosis through increasing the amount of Ca^2+^ and downregulating the expression of Bcl-2. Meanwhile, the level of Fas death receptor was elevated which indicated that AECK could lead to exogenous apoptosis in U937. The expressions of oncogene c-Myc and c-Fos were suppressed which manifested that AECK could negatively regulate the growth, proliferation, and tumorigenesis of U937 cells. This research presented the primary antitumor activity of AECK which would contribute to the widely use of* Cordyceps kyushuensis* Kob as a functional food and medicine.

## 1. Introduction

With the increasing prevalence of cancer diagnoses and mortality rates, cancer becomes the leading cause of mortality since 2010 in China [1]. Despite the remarkable advances in cancer therapy, a low survival rate is still a burden to society. Up to now, studies have shown that cancerous cells could be wiped out by antineoplastic drugs through inducing apoptosis [2, 3], which provides effective method and long-term perspective to cure cancer.

Apoptosis, which can eliminate the unwanted cells in organism, plays an important role in maintaining the development and health of human body [4]. Two main signaling pathways, the extrinsic pathway and the intrinsic pathway, are involved in apoptosis [5]. When cell surface receptors are stimulated by bounding to special ligands, the extrinsic pathway is mediated. With death receptors trimerized, procaspase 8 is activated to stimulate effector caspases, resulting in apoptosis finally [6]. The intrinsic pathway is the mitochondrial medicated pathway, which is characterized as increased mitochondrial permeability and release of cytochrome c [7]. Meanwhile, the c-myc and c-fos oncogenes are pivotal to the genesis of many human cancers. That c-fos is a member of Fos family, which can form the group of AP-1 protein with Jun family member. AP-1 complex could participate in regulating the proliferation, angiogenesis, and metastasis of tumor cells [8]. The proto-oncogene myc lies at the crossroads of many growth promoting signal transduction pathways and is an immediate early response gene downstream of many ligand-membrane receptor complexes [9].

*Cordyceps* genus is an important kind of entomogenous fungi which includes over 500 species; the famous traditional Chinese medicine* Cordyceps sinensis* and* Cordyceps militaris *both belong to it.* Cordyceps* has long been demonstrated to possess many bioactive components, such as cordycepin, cordycepic acid, and Cordyceps polysaccharides. All of them show various pharmacological activities, for example, antiaging, antitumor, antioxidant, anti-inflammatory, and immunomodulatory. Cordycepin, which was first isolated from* C. militaris* in 1949 [10], is an analogue of 3′deoxyadenosine. Compared to adenosine, cordycepin lacks a 3′hydroxyl group, which increases its potency [11]. According to previous studies, cordycepin could give play to antineoplastic activity through interfering with purine biosynthesis and DNA/RNA synthesis [12]. In addition, cordycepin also could induce apoptosis [13], cell cycle arrest [14], antimetastasis, inhibition of platelets aggregation, and antitrypanosomiasis [15]. Except for cordycepin, Cordyceps polysaccharides are a series of important compounds which compose 3%–8% total weight of Cordyceps and have shown a wide range of different bioactivities according to its molecular weight and characteristics, such as antitumor [16], anti-influenza virus, immunopotentiation [17], and antioxidant effects [18].

As is known to all,* C. sinensis *has long been used as functional food and tonic to treat many illnesses for centuries [19], especially in East Asia. With the realization of its medicinal and health value, the supply of* C. sinensis *is inadequate for the demand because of its low yield in high-altitude areas where it cannot be easily harvested [20]. Meanwhile, recent reports illustrate that* C. sinensis*' inability to produce cordycepin was due to a lack of cordycepin biosynthesis related genes. However,* C. militaris*,* A. nidulans*, and* C. kyusyuensis* have been reported to have related genes to produce cordycepin [21].* C. kyushuensis* in this manuscript is referred to as the abovementioned* C. kyusyuensis*. In 2008, Dai et al. revised the checklist of medicinal fungi in China, and* Cordyceps kyushuensis* was renamed* Cordyceps kyusyuensis* [22]. But in recent years, the two nomenclatures of* Cordyceps kyushuensis* and* Cordyceps kyusyuensis* are both used, so in this manuscript, the authors still use the Latin name “*Cordyceps kyushuensis*.”* C. kyushuensis *Kob is the only species of* Cordyceps *which infects the* Clanis bilineata * Walker ever reported in China [23], and it stands a chance of being a kind of substitute of* C. sinensis*. After years of work, artificial culturing of* C. kyushuensis* Kob has successfully laid a foundation of producing the substance in large scale operations. Therefore, it is necessary to make a further research on its medicinal potential and function mechanism, which may lead to the discovery and development of a new medical resource.

Decocting traditional Chinese medicine to extract effective ingredients by boiling in water is a common way to take medicine in folk. Meanwhile, a large body of studies reported that there are lots of active ingredients including cordycepin in aqueous extracts of* C. militaris *which could induce apoptosis to cure cancer [24]. It was investigated that* C. kyushuensis *Kob has similar effective ingredients with* C. militaris*, which implies that studying the aqueous extracts of* C. kyushuensis *Kob (AECK) is valuable in practice and has an extensive application. In the present study, the cytotoxic effect of AECK was investigated on human tissue lymphoma cell lines (U937) and human nonadherent myelogenous leukemia cell lines (K562), and the results indicated that AECK can inhibit the proliferation of these two cell lines and induce apoptosis through intrinsic and extrinsic signal pathway. Meanwhile, AECK can also downregulate the expressions of oncogene c-fos and c-myc which suppress the progression of carcinoma.

## 2. Materials and Methods

### 2.1. Strain, Reagents and Antibodies

Wild* C. kyushuensis* Kob was obtained from Mount Meng, Shandong Province. Cordycepin was purchased from Sigma (St. Louis, MO, USA). Antibodies Bcl-2, c-Fos, and c-Myc were purchased from BD (BD, USA). Antibodies Fas were purchased from Pharmingen (USA).

### 2.2. Preparation and Component Analysis of AECK

Aqueous extracts of* C. kyushuensis* Kob (AECK) was prepared by ultrasonic extraction of* C. kyushuensis* Kob fruiting body with distilled water. Soak 5.0g* C. kyushuensis* Kob power into 50 ml distilled water for 5 h, and then use ultrasound for 5s, interval for 5s, 120 times with 400W, and centrifuge at 10000 rpm for 10 min, finally receiving supernate as standby application. Component analysis of AECK was performed using Capillary Zone Electrophoresis (CZE) with separation voltage 20 kV, detection wavelength 258 nm, sample size 0.5psi, injection time 5 sec, column temperature 20°C, and 0.025 mol/L borax solution as buffer solution.

### 2.3. Cell Lines and Cell Culture

Human tissue lymphoma cell lines (U937) and human nonadherent myelogenous leukemia cell line (K562) were provided by Institute of Hematology and Blood Diseases Hospital, Chinese Academy of Medical Sciences. RPMI 1640 medium and fetal bovine serum (FBS) were purchased from Gibco (Grand Island, NY). Cells were cultured with RPMI 1640 medium containing 5% FBS in a humidified incubator containing 5% CO_2_ at 37°C.

### 2.4. MTT Assay and Measurement of IC_50_

1 × 10^4^ cells/well were seeded in 96-well plates and disposed of increasing concentrations of cordycepin and AECK. After 48 h treatment, MTT working solution was added to 96-well culture plates and incubated continuously at 37°C for 3 h. Discard the culture supernatant and added DMSO to dissolve the formazan crystals. ELISA reader (Molecular Devices, Sunnyvale, CA) was used to measure the absorbance of each well at 540 nm.

100 *μ*L/well increasing concentrations of AECK and 100 *μ*L/well nutrient solution were disposed of in 96-well plates, 200 *μ*L/well in total. Using the method of simple interpolation to calculate IC_50_

(1) the  drug  concentration  which  was  greater  than  50%  inhibition  rate  -  XX  -  drug  concentration  which  was  less  than  50%  inhibition  rate=the  inhibition  rate  which  was  greater  than  50%  -  X50−the    inhibition  rate  which  was  less  than  50%

### 2.5. Determination of Caspase Activity

The Caspase activities were determined by colorimetric assays using Caspase-3 activation kits (Thermo Fisher Scientific, USA). The synthetic tetrapeptides in the kits used were labeled with p-nitroaniline. Firstly, the cells were lysed in the supplied lysis buffer. The lysate was gathered and incubated with the supplied reaction buffer containing dithiothreitol (DTT) and substrates at 37°C. The absorbance at 405 nm was measured by ELISA reader to determine caspase activity.

### 2.6. Hoechst 33342 Staining

Morphology of apoptotic nuclear was measured by Hoechst 33342 staining. Cells were seeded in 96-well plates at a density of 1 × 10^4^ cells/well. After incubation, cells were treated with 64 *μ*g/mL AECK for 48 h. After treatment, 100 *μ*L Hoechst 33342 (2 *μ*g/mL) was added to each well and incubated for at least 15 min before imaging. At last, apoptotic nuclear morphology was observed with fluorescent microscope [25].

### 2.7. Flow Cytometric Analysis of Apoptosis Related Protein

Cells treated with AECK were harvested, washed with PBS, fixed with ice-cold 70% ethanol, washed with PBS, and then added PBS to a constant volume of 100 *μ*L. The cells were incubated with primary antibodies for 20 min at room temperature, and then 10 *μ*L FITC-secondary antibodies were added, incubated in dark for 20 min, and washed with PBS. Flow cytometry analyses were carried out using a flow cytometer (FACS Calibur; Becton Dickinson, San Jose, CA). Cell-Quest Plot software was used to analyze the data.

## 3. Results

### 3.1. Component Analysis of AECK

**Figure 1:**
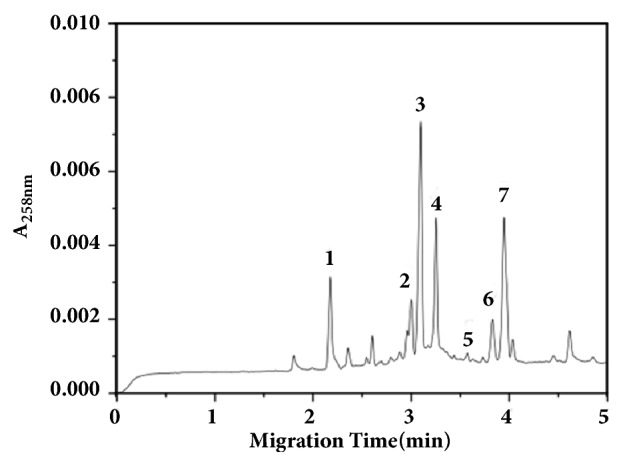
CZE map of the stroma of* C. kyushuensis* 1. Cordycepin. 2. Adenine. 3. Uracil. 4. Adenosine. 5. Hypoxanthine. 6. Guanosine. 7. Uridine.

Injecting samples in the selected condition described above to detect chromatographic peaks of related components, the results showed that wild* C. kyushuensis* Kob includes cordycepin, adenosine, guanosine, uridine, uracil, thymine and tiny hypoxanthine, and deoxyuridine (Figure 1). Cordycepin content of Wild* C. kyushuensis *Kob was 1.017 mg/g.

### 3.2. AECK Inhibited the Proliferation and Viability of U937 and K562 Cells

**Figure 2:**
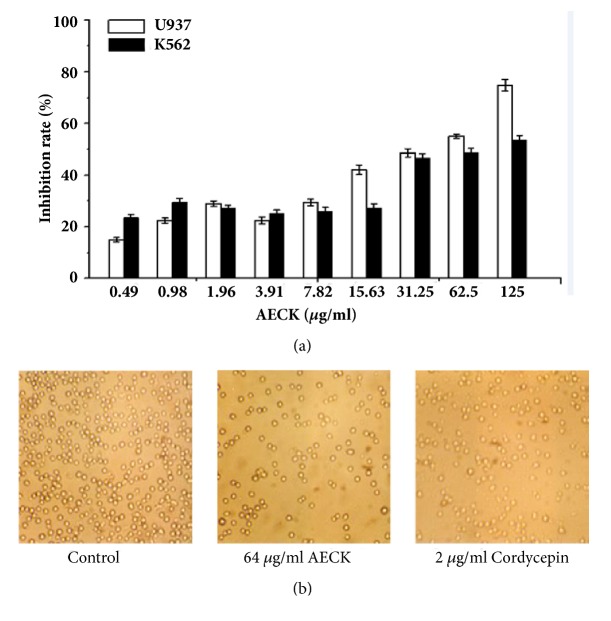
AECK could inhibit cell proliferation and viability of U937 and K562 (a) U937 and K562 were treated with increasing concentrations of AECK for 48 h, and the viability was measured by MTT. (b) The observation of U937 by optical microscope after treated by AECK (64 *μ*g/ml) and cordycepin (2 *μ*g/ml) for 48 h.

To test the cytotoxic effect of AECK on the proliferation of U937 and K562, the cells were treated with different concentrations of AECK and then cell viability was measured by the MTT assay. As shown in Figure 2, the viability of U937 and K562 cells was significantly decreased after being exposed to AECK for 48 h in a concentration-dependent manner, and the IC_50_ values of U937 and K562 were 31.23 *μ*g/ml and 62.5 *μ*g/ml, respectively.

### 3.3. AECK Induced Cell Apoptosis in U937 and K562 Cells

**Figure 3:**
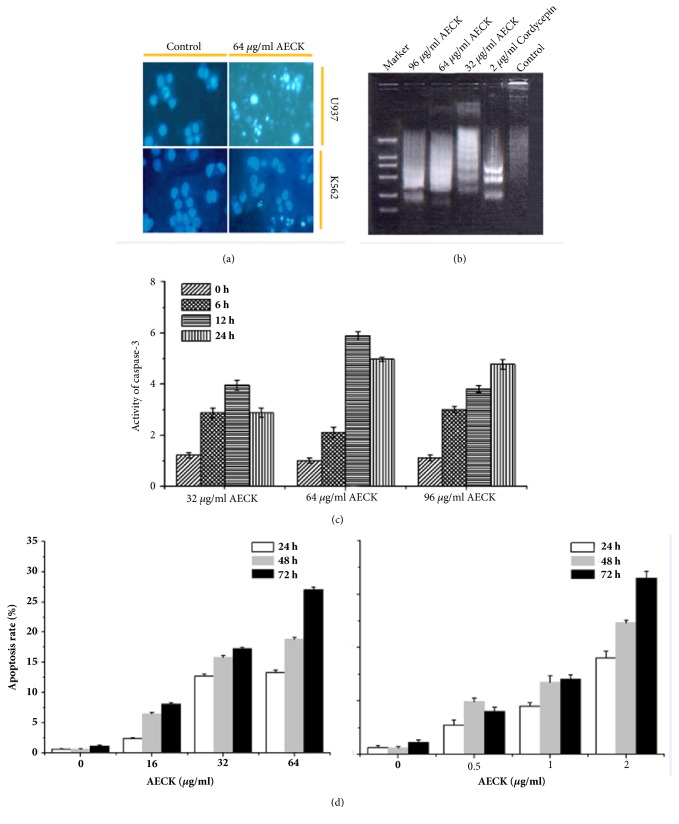
AECK induced apoptosis in U937 and K562 (a) U937 and K562 were exposed to 64 *μ*g/mL AECK for 48 h, and then Hoechst 33258 staining was used to detect apoptosis. (b) To analyze the DNA fragmentation, the cells were treated with the indicated concentrations of AECK for 48 h, and DNA was extracted, resolved in 1.5% agarose gel, and then visualized using ethidium bromide, and 2 *μ*g/ml cordycepin was used to be positive control; (c) U937 was disposed of in different concentrations of AECK and cordycepin. The extracts from cells were assayed for caspase-3 activity by using colorimetric assay. (d) Flow cytometry analysis of U937 after treatment by increasing concentration of cordycepin and AECK for indicated time period.

To observe whether AECK induces apoptosis in U937 and K562, cell morphology was analyzed with Hoechst33258 Staining. Results showed that U937 and K562 cells presented cell shrinkage, chromatin condensation, and cytoplasmic blebbing after treatment with AECK for 48 h which were characteristic of apoptosis (Figure 3(a)). DNA fragmentation was also a main feature which could be characterized as apoptosis and was often detected by DNA ladder experiment.  Figure 3(b) showed that treatment with AECK caused DNA fragmentation. And the DNA fragments aroused by higher concentration of AECK tended to be shorter. Cordycepin was used as positive control.

Caspases are very important mediators of apoptosis and can contribute to the general apoptotic morphology through the cleavage of various cellular substrates. Caspase-3 activity assays were performed to test U937 and K562 cell apoptosis after treatment with AECK. As shown in Figure 3(c), the fact that treatment of AECK (32 *μ*g/mL or 64 *μ*g/mL) for 6 h could significantly increase caspase-3 activity. When further treated with 32 *μ*g/mL and 64 *μ*g/mL AECK, the results showed that caspase-3 activity increased 3.78 times and 5.82 times for 12 h, respectively, and 2.78 times, 4.76 times for 24 h, respectively. To investigate whether AECK could induce apoptosis, different concentrations of AECK and cordycepin were used to treat U937 to test apoptosis rate. According to Figure 3(d), it shows that, with the increase of concentration and time, the apoptosis rate increased in a concentration and time dependent manner. When treated with 64 *μ*g/mL AECK for 72 h, the apoptosis rate was up to 27.04%. At the same time, 2 *μ*g/mL cordycepin treated U937 for 72 h and its apoptosis rate was 16.52%.

### 3.4. AECK Induced Apoptosis in Both Extrinsic and Intrinsic Pathways

**Figure 4:**
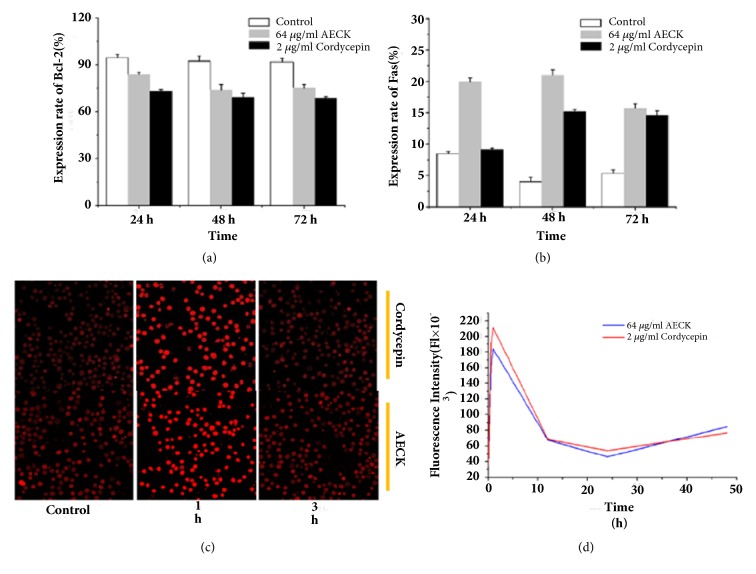
AECK extract induces apoptosis in both extrinsic and intrinsic pathways. (a) Antibodies Bcl-2 and (b) Fas were utilized to incubated with U937 cells and then treated with FITC-conjugated anti-rabbit IgG, analyzed with flow cytometry; (c) U937 was seeded on cells climbing, treated with 64 *μ*g/ml AECK and 2 *μ*g/ml cordycepin for various lengths of time, And then analyzed by confocal fluorescent microscopy after stained with Fluo-3AM. (d) The line chart of Ca2+ density variation according to indicated time.

To further study how AECK induced apoptosis in U937 cells, the expression of Bcl-2 and Fas was observed in protein levels. Results of flow cytometry showed that Bcl-2 could be downregulated after being treated with AECK in a time dependent manner; the relative expressions were 83.88%, 74.02%, and 75.22%, in contrast to the negative control which was 94.53%, 92.38%, and 91.81% after being treated for 24 hours, 48 hours, and 72 hours. While a significant upregulation of Fas could be observed in a time dependent manner too, the relative protein expressions of it were 19.94%, 20.98%, and 15.72%, in contrast to the negative control which was 8.47%, 4.02%, and 5.35% after being treated in 24 hours, 48 hours, and 72 hours (Figures 4(a) and 4(b)). When intrinsic apoptosis was induced, endoplasmic reticulum could release Ca^2+^, which could trigger mitochondria to release cytochrome C. In order to detect the concentration of Ca^2+^, using Fluo-3AM to stain Ca^2+^ and observed with Laser Scanning Confocal Microscope. As shown in Figures 3(c) and 3(d), an obvious increase of fluorescence intensity could be observed after treatment of AECK (64 *μ*g/mL) for 1 h, which indicated that the concentration of Ca^2+^ in U937 went up rapidly. It is a remarkable fact that being treated for longer time, the fluorescence intensity began to attenuate and the concentration of Ca^2+^ declined in 12 h and 24 h. One thing to be noted is that the concentration of Ca^2+^ raised again after 48 h.

### 3.5. AECK Downregulates the Expression of Oncogenes c-Myc and c-Fos

**Figure 5:**
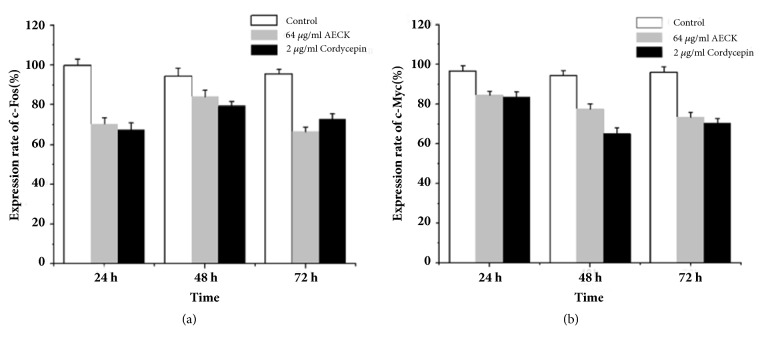
AECK downregulates the expression of oncogene c-Myc and c-Fos. U937 were treated with (a) c-Fos and (b) c-Myc antibodies and afterwards incubated with FITC-conjugated anti-rabbit IgG and subsequently analyzed with flow cytometry.

In consideration of the important role of c-Myc and c-Fos in carcinoma progression, flow cytometry was performed to detect its conditions of expression. As shown in Figures 5(a) and 5(b), the positive rates of c-Fos protein expression were 70.34%, 84.13%, and 66.49% after treatment of 64 *μ*g/mL AECK in 24 h, 48 h, and 72 h, while the positive rate of c-myc protein expressions were 84.47%, 77.62%, and 73.49% after treatment of 64 *μ*g/mL AECK in 24 h, 48 h, and 72 h. The results showed that the treatment of AECK (64 *μ*g/mL) in U937 cells could suppress the expression of c-myc and c-Fos in a time dependent manner.

## 4. Discussion

As is known to all,* C. sinensis* as a famous traditional Chinese medicine has been used for centuries, especially in East Asia. The ever-growing market demand is leading to overharvesting of* C. sinensis*, causing severe devastation of gragile Alphine environments. To meet the requirement of market and protect our environments, people find suitable substitutions all the time. Meanwhile Wang et Al. [21] reported that* C. sinensis* has no ability to produce cordycepin, because there were no cordycepin biosynthesis related genes.* C. kyushuensis* Kob which was found in Mount Meng area, Shandong Province, has been demonstrated to have lots of pharmacological active components including cordycepin. Cordycepin which has been reported to possess various pharmacological effects, such as anticancer, antiviral, antiaging, and anti-inflammation, has been demonstrated to be a potential source of antitumor drugs. Using water to boil traditional Chinese medicine is a common way to take medicine in China, so investigating the aqueous extracts of* C. kyushuensis* is very realistic significance. In this research, AECK was prepared by ultrasonic extraction and Capillary Zone Electrophoresis (CZE) was used to analysis the compositions of AECK. The CZE result showed that there are cordycepin, adenosine, guanosine, uridine, uracil, thymine and tiny hypoxanthine, and deoxyuridine in it. Cordycepin content of Wild* C. kyushuensis* Kob was 1.017 mg/g. The result of MTT assay demonstrated that AECK could inhibit the proliferation in U937 and K562 cells, and the IC_50_ values of U937 and K562 were 31.23 *μ*g/mL and 62.5 *μ*g/mL, respectively.

Apoptosis, type I programmed cell death, which could be recognized by various morphological characteristics, was induced by AECK after 48 h treatment in U937 and K562 cells. Chromatin condensation was observed by Hoechst 33258 staining in U937 and K562. Moreover, DNA ladder experiment indicated that treatment of AECK led to DNA fragmentation. The increase of Caspase 3 activity showed that AECK triggered Caspase dependent apoptosis and the apoptotic rate examined by flow cytometry demonstrated that AECK induced apoptosis both a time and a dose dependent manner in U937 cell. According to results above, AECK could induce caspase dependent apoptosis in U937. Mitochondria involved intrinsic apoptosis and death receptors mediated external apoptosis have been reported to be two main apoptosis pathways in most cancer cells. Fas is a transmembrane protein belonging to the tumor necrosis factor receptors (TNF) superfamily and the death receptor subfamily [26]. Fas can transduce signals that lead to external apoptotic cell death[27]. In this study, the upregulation of Fas indicated that AECK triggered apoptosis in U937 through Fas mediated exogenous pathway. Bcl-2, an antiapoptosis protein which belongs to Bcl-2 family of proteins, has been considered as a potential target in cancer treatment [28]. Treatment of AECK inhibited the expression of Bcl-2 and triggered apoptosis in U937 cell. The level of Ca^2+^ in cytoplasm was elevated which showed that intrinsic apoptosis was induced. Release of Ca^2+^ from endoplasmic reticulum revealed that ER stress may be involved in apoptosis, while further study was still needed to confirm this hypothesis [29].

The c-myc and c-fos oncogenes play important role in the formation of many tumor cells. Downregulation of c-fos may prevent the formation of Jun family and then inhibited the proliferation, angiogenesis, and metastasis of cancer cells. Suppression of c-myc may be likely to disorganize the signaling pathways that promote the growth of cancer cells which also affect the proliferation, tumorigenesis of tumor cells.

In summary, the results indicated that AECK could inhibit the proliferation of U937 and K562, trigger mitochondria dependent intrinsic apoptosis, and regulate external apoptosis in U937. Moreover, AECK could inhibit the proliferation, tumorigenesis of tumor cells through downregulating the expression of c-fos and c-myc. Through this study, it is believed that AECK could contribute to the treatment of cancer and widely use of* C. kyushuensis* Kob to carry forward the traditional Chinese medicine and healthcare fields.

## Data Availability

All data was provided in the article and there are no more data to be uploaded.
